# Prevalence and virulence gene profiling of enteroaggregative *Escherichia coli* in malnourished and nourished Brazilian children

**DOI:** 10.1016/j.diagmicrobio.2017.06.024

**Published:** 2017-10

**Authors:** Alexandre Havt, Ila FN Lima, Pedro HQS Medeiros, Marco AF Clementino, Ana KS Santos, Marília SMG Amaral, Herlice N. Veras, Mara MG Prata, Noélia L. Lima, Alessandra Di Moura, Álvaro M. Leite, Alberto M. Soares, José Q. Filho, Eric R. Houpt, James P. Nataro, Richard L. Guerrant, Aldo AM Lima

**Affiliations:** a Institute of Biomedicine for Brazilian Semiarid, Federal University of Ceará, 1315 Coronel Nunes de Melo, 60430-270, Fortaleza, Brazil; b Institute for the Promotion of Nutrition and Human Development, 15 Professor Carlos Lobo, 60281-740, Fortaleza, Ceará, Brazil; c Center for Global Health & Division of Infectious Diseases and International Health and Department of Pediatrics, University of Virginia, 1400 W Main Street, 22908-1379, Charlottesville, VA, USA

**Keywords:** Malnutrition, Enteroaggregative *E. coli*, Virulence profile

## Abstract

The impact of enteroaggregative *E. coli* (EAEC) infection on childhood malnutrition and inflammation has been suggested, regardless of diarrhea. We investigated whether EAEC and its virulence-related genes (VRGs) are associated with malnutrition in a case–control study. Children aged 6–24 months from Brazil were enrolled as malnourished if weight-for-age Z-score (WAZ) ≤ −2 and nourished if WAZ > −1. Stools were cultured and examined for *E. coli*. DNA was extracted from fecal isolates and tested for EAEC by polymerase chain reaction (PCR). Positive samples were analyzed by 5 multiplex PCRs to identify 20 EAEC VRGs. Biomarkers of intestinal barrier function and inflammation were measured. The prevalence of EAEC was 39.94%. Samples that presented both *aaiC* and *aatA* genes were associated with malnutrition (*P* = 0.045). A high prevalence of VRGs was observed and the *aafC* gene was significantly associated with malnourished (*P* = 0.0101). Strains lacking *aar* and *pic* genes were associated with malnutrition (*P* = 0.018), while the concomitant presence of *aar*, *pic*, *agg4A*, and *capU* genes was associated with nourished (*P* = 0.031). These data reinforce the EAEC impact on malnutrition, the importance of *aar* as negative regulator and the great contribution of AAF/II fimbria for the pathobiology of EAEC.

## 1 Introduction

Enteroaggregative *Escherichia coli* has been cited as an important childhood enteric pathogen worldwide (Jensen et al., 2014). A recent large and lethal German outbreak caused by a Shiga toxin (Stx)-producing EAEC, brought attention to this pathogen (Scheutz et al., 2011). However, EAEC causes only a subclinical enteric infection or gut colonization in some subjects (Bueris et al., 2007, Lima et al., 2013). Recent large multi-center studies evaluating children from low and middle-income countries did not find association between EAEC and diarrhea (Kotloff et al., 2013).

Previous analyses of children from a Brazilian shantytown presenting with EAEC infection without diarrhea demonstrated that they had evidence of intestinal inflammation, in addition to significant growth impairment (Steiner et al., 1998). The association of EAEC colonization with height-for-age Z-score (HAZ) has been recently also shown in Panamanian children (Gutiérrez et al., 2014), highlighting the contribution of EAEC to childhood stunting regardless of the presence of diarrhea.

Several studies have associated different virulence-related genes (VRGs) of EAEC with diarrhea outcome (Bafandeh et al., 2015, Lima et al., 2013, Nuesch-Inderbinen et al., 2013), and the high heterogeneity of EAEC strains is a major contributor for this phenomenon (Estrada-Garcia and Navarro-Garcia, 2012). A great variety of VRGs is evaluated in the studies, including genes related to adherence factors, proteases, toxins, metabolic enzymes and other regulator factors (Boisen et al., 2012).

Despite the great number of EAEC VRGs described and their extensive investigation on strains associated with diarrhea, there is few data about association of EAEC infection with different clinical outcomes as malnutrition. Our main goal in this work is to define the importance of EAEC as a pathogen impairing nutritional status of children from an urban area of Brazil and to identify specific traits of EAEC VRGs that would be associated with malnutrition. In addition, we further investigated potential host biomarkers of inflammation and intestinal function correlated with specific malnutrition-associated EAEC strains.

## 2 Materials and methods

### 2.1 Geographic location, ethical approval, and study design

This study was conducted in Fortaleza, the capital city of the State of Ceará, located in the Northeast of Brazil at the Institute for the Promotion of Nutrition and Human Development (IPREDE) in collaboration with the Institute of Biomedicine, Faculty of Medicine, Federal University of Ceará. This case–control study was part of the Malnutrition-Enteric Diseases (MAL-ED) network, as described by Lima et al. (2014). The protocol and consent form were approved by the local institutional review board (IRB) at the Federal University of Ceará, the national IRB Conselho Nacional de Ética em Pesquisa (CONEP, #15701), and the University of Virginia in the United States.

The case–control study compared malnourished and nourished children aging 6–24 months who were enrolled from August 2010 to June 2013. Malnourished were defined as moderate to severely underweight children, determined by weight-for-age Z-score (WAZ) less than −2. For this manuscript, age, sex, and neighborhood-matched nourished were enrolled following the criteria of WAZ higher than −1. Both groups did not present any specific illness or fever. All children that their mother or primary care-giver with legal custody have had given informed consent to participate in the study and met the entry criteria were quarterly followed-up for one year. Children who required prolonged hospitalization or presented serious health issues; or had parents/caregivers with cognitive deficits or less than 16 years old were excluded from the study. However, after approving the participation of his or her children by signing the consent form, each parent or responsible guardian was asked to return 10 days after enrollment with a stool sample and for anthropometric measurements.

### 2.2 Microbiologic assays

The stools of each child (n = 313) were used in this study to detect pathogenic *Escherichia coli*. Stools were cultured in MacConkey agar for up to 48 hours and plates were examined for flat, lactose fermenting colonies. Colonies that morphologically resembled *E. coli* were tested for their ability to convert tryptophan into the indole. If positive, 5 colonies were pooled for DNA extraction. When there were multiple *E. coli*-like colony morphologies, we had chosen five that had the most variety of morphotypes (Taniuchi et al., 2012). The procedures were performed as previously described (Houpt et al., 2014).

DNA from a pool of up to 5 *E. coli* strains was isolated by boiling method (Boisen et al., 2012). A polymerase chain reaction (PCR) described elsewhere (Houpt et al., 2014) was used to diagnose 5 strains of diarrheagenic *E. coli* (EAEC, enteropathogenic *E. coli* – EPEC, enterohemorrhagic *E. coli* – EHEC, enterotoxigenic *E. coli* – ETEC and enteroinvasive *E. coli* – EIEC) using 9 associated virulence genes. Primers specific for EAEC identification were *aaiC* and *aatA*. Samples were considered positive for EAEC if we could detect either one of the 2 diagnostic genes or both. Based on this definition, 3 distinct patterns were used to explore if there was any association among malnourished or nourished: (i) samples positive only for *aaiC*; (ii) samples positives only for *aatA*; (iii) samples positives for both genes (*aaiC* and *aatA*).

Only EAEC positive samples were further analyzed by multiplex PCRs to identify 20 EAEC VRGs, adapted from Lima et al. (2013) (Table 1). The multiplex PCR conditions were one cycle for 15 min at 95 °C; 35 cycles for 45 s at 95 °C, 45 s at 57 °C and 1,25 min at 72 °C; and an extension step for 10 min at 72 °C in a MyCycler™ thermal cycler (BioRad Laboratories). Electrophoresis of ethidium bromide-stained 2% agarose gels in 1× Tris-acetate-EDTA buffer was performed, and bands were visualized and photographed using ChemiDoc XRS (BioRad Laboratories).

**Table 1:** Description of genes, GenBank accession numbers, primer sequences, and size of the obtained products of the genes used for diagnosis of enteroaggregative *Escherichia coli* and its related virulence genes.

Description of the target genes (GenBank accession No.)	Type of PCR	Primer sequence (5′ → 3′)	Size (bp)
Diagnostic genes			
*aaiC* – *aggR*-activated island (FN554766.1)	MAL-ED Multiplex	ATTGTCCTCAGGCATTTCAC ACGACACCCCTGATAAACAA	215
*aatA* – anti-aggregation protein transporter (AY351860)		CTGGCGAAAGACTGTATCAT CAATGTATAGAAATCCGCTGTT	630

Virulence genes			
*astA* – aggregative heat-stable toxin A, EAST1 (L11241)	Multiplex 1	ATGCCATCAACACAGTATAT GCGAGTGACGGCTTTGTAGT	110
pet – plasmid-encoded toxin (AF056581)		GGCACAGAATAAAGGGGTGTTT CCTCTTGTTTCCACGACATAC	302
*sigA* – Shigella IgA-like protease homolog (NC_004337)		CCGACTTCTCACTTTCTCCCG CCATCCAGCTGCATAGTGTTTG	430
*pic* – protein involved in colonization (AF097644)		ACTGGATCTTAAGGCTCAGGAT GACTTAATGTCACTGTTCAGCG	572
*sat* – secreted autotransporter toxin (AE014075)		TCAGAAGCTCAGCGAATCATTG CCATTATCACCAGTAAAACGCACC	932
*orf3* – cryptic protein (FN554767.1)	Multiplex 2	CAGCAACCATCGCATTTCTA CGCATCTTTCAATACCTCCA	121
*aap* – anti-aggregation protein, Dispersin (Z32523)		GGACCCGTCCCAATGTATAA CCATTCGGTTAGAGCACGAT	250
*agg3A* – AAF/III fimbrial subunit (AF411067)		CCAGTTATTACAGGGTAACAAGGGAA TTGGTCTGGAATAACAACTTGAACG	370
*sepA* – Shigella extracellular protease (Z48219)		GCAGTGGAAATATGATGCGGC TTGTTCAGATCGGAGAAGAACG	794
*eilA* – Salmonella HilA homolog (FN554766.1)	Multiplex 3	AGGTCTGGAGCGCGAGTGTT GTAAAACGGTATCCACGACC	130
*aafA* – AAF/II fimbrial subunit (AF012835)		CTACTTTATTATCAAGTGGAGCCGCTA GGAGAGGCCAGAGTGAATCCTG	289
*agg3/4C*⁎ – Usher, AAF/III-IV assembly unit (AF411067, AB255435, EU637023)		TTCTCAGTTAACTGGACACGCAAT TTAATTGGTTACGCAATCGCAAT TCTGACCAAATGTTATACCTTCAYTATG	409
*aafC* – Usher, AAF/II assembly unit (AF114828)		ACAGCCTGCGGTCAAAAGC GCTTACGGGTACGAGTTTTACGG	491
*aar (previously called orf61*)- negative regulator of *aggR* (FN554767.1)	Multiplex 4	AGCTCTGGAAACTGGCCTCT AACCGTCCTGATTTCTGCTT	108
*aggA* – AAF/I fimbrial subunit (Y18149, AY344586)		TCTATCTRGGGGGGCTAACGCT ACCTGTTCCCCATAACCAGACC	220
*capU* – Hexosyltransferase homolog (AF134403)		CAGGCTGTTGCTCAAATGAA GTTCGACATCCTTCCTGCTC	395
*air* – Enteroaggregative immunoglobulin repeat protein (FN554766.1)		TTATCCTGGTCTGTCTCAAT GGTTAAATCGCTGGTTTCTT	600
*agg4A* – AAF/IV fimbrial subunit (EU637023)	Multiplex 5	TGAGTTGTGGGGCTAYCTGGA CACCATAAGCCGCCAAATAAGC	169
shiA – shiA-like inflammation suppressor (ECB_03517)		CAGAATGCCCCGCGTAAGGC CACTGAAGGCTCGCTCATGATCGCCG	292
*aggR* – transcriptional activator (Z18751)		GCAATCAGATTAARCAGCGATACA CATTCTTGATTGCATAAGGATCTGG	426

NOTE. The multiplex PCR conditions were one cycle for 15 min at 95 °C; 35 cycles of 95 °C for 45 s, 57 °C for 45 s and 72 °C for 1,15 min; and an extension step for 10 min at 72 °C.

⁎ Two forward primers and 1 reverse primer were used for the amplification of agg3/4C. This primer set was designed to amplify the usher gene from both AAF/III and IV.

Another pool of DNA, extracted by the same boiling method mentioned above, of EAEC strains were used as positive control in each of the 5 performed multiplexes. For the multiplex 1 we used DNA from the EAEC strains 042 (*astA*, *pet* and *pic* genes), H223-1 (*sigA* and *pic* genes), JM221 (*sat* and *pic* genes) and 239-1 (*sat* and *pic* genes). Multiplex 2 had DNA from the positive control strains 042 (*aap* and *orf3* genes), H223-1 (*sepA* gene) and 55989 (*agg3A* gene). The DNA from strains 042 (eilA, *aafC*, *aafA* genes) and 55989 (*aag3/4C* gene) were used as positive sample for multiplex 3, but DNA from JM221 (*aggA* gene) and 042 (*aar*, *air* and *capU* genes) were used for multiplex 4. Finally, multiplex 5 were performed using DNA from JM221, 042 (*aggR* and *shiA* genes) and H223-1 (*aag4A* gene) as positive strains.

### 2.3 Host biomarkers assessment

Selected host biomarkers of intestinal barrier function, intestinal and systemic inflammation were measured in stool (lactoferrin, alpha-1-antitrypsin, myeloperoxidase, neopterin and regenerating gene 1β – REG1B), urine (lactulose/mannitol ratio) and serum samples (serum amyloid A – SAA, intestinal fatty acid-binding protein – I-FABP, lipopolysaccharide binding protein – LBP, soluble form of CD14 – sCD14, calprotectin and endotoxin core antibody -ENDOCAB). Specimens were collected and frozen at -80 °C pending processing.

The lactulose/mannitol (L/M) ratio test was performed as described previously (Barboza et al., 1999). Enzyme-linked immunosorbent assay (ELISA) kits for serum substances were purchased from Hycult Biotech (Uden, Netherlands), while lactoferrin, alpha-1-antitrypsin, myeloperoxidase, neopterin and REG1B kits for stool samples were purchased from TechLab (Blacksburg, United States), Immuchrom (Heppenheim, Germany), Immundiagnostik (Bensheim, Germany), Genway Biotech (San Diego, CA) and TechLab (Blacksburg, VA), respectively. All procedures were performed according to manufacturer instructions.

### 2.4 Data analysis

Fisher's exact and odds ratio tests were used to compare data derived from case and control children regarding the presence of virulence genes. To investigate the correlation of specific combinations of VRGs and of co-pathogens with malnutrition, we employed classification and regression tree (CART; Salford Systems, San Diego, CA) analysis, which constructs a model in stepwise fashion and the outcome shows a combination of factors most strongly associated with malnourished or nourished. For the VRGs analysis, CART was performed inputting 20 VRGs of interest as binary (present/absent) independent predictive variables, whereas for the co-infection analysis a large list of pathogens prevalence was inputted (please see the list and detection methodologies on Houpt et al., 2014). Case–control status was the binary dependent outcome variable. For the analysis of biomarkers results, Mann–Whitney *U* tests were performed evaluating median differences between case and control groups. Statistical analyses were performed using GraphPad Prism (GraphPad software, version 5.01). *P*-values <0.05 were considered statistically significant.

## 3 Results

### 3.1 Characteristics of the study population and EAEC prevalence

Among the 313 children, 152 (48.56%) were malnourished and 161 (51.44%) were nourished. More than 3 quarters of these families (80.17%, 186/232) had a monthly income below US $285 (R $1000.00). Regarding the child's diarrhea status, 5.43% (17/313) of the total samples were diarrheal – 3.95% (6/152) from malnourished and 6.83% (11/161) from nourished. There was no significant association between case definition and presence of diarrhea (*P* = 0.3186).

Among total population, 39.94% (125/313) of the children were positive for EAEC. Of those, 52.80% (66/125) were defined as malnourished and 47.20% (59/125) were nourished. Samples that presented both the diagnostic genes (*aaiC* and *aatA*) were more prevalent (56.06%, 37/66) and associated with malnourished (*P = 0.045, OR = 1.84, 95% CI 1.03–3.25) (Table 2). Overall, there was no association between EAEC positive diagnosis and presence of diarrhea (*P* = 0.4488).

**Table 2:** Prevalence of enteroaggregative *Escherichia coli* diagnostic genes among malnourished and nourished.

	Malnourished (n = 152)	Nourished (n = 161)	Total (n = 313)	*P* value	Odds ratio	95% IC
	No. (%)	No. (%)	No. (%)			
*aaiC* only^1^	10 (6.58)	9 (5.59)	19 (6.07)	0.814	1.19	0.47–3.01
*aatA* only^1^	19 (12.50)	26 (16.15)	45 (14.38)	0.421	0.74	0.39–1.40
*aaiC* + *aatA*	37 (24.34)	24 (14.91)	61 (19.49)	0.045	1.84	1.03–3.25
Total	66 (43.42)	59 (36.65)	125 (39.94)			

1 *aaiC* = *aggR*-activated island; *aatA* = anti-aggregation protein transporter.

Table 3 describes comparison analysis of characteristics from malnourished and nourished study population positive for EAEC, regarding age, sex, birth weight, head circumference and anthropometric and socioeconomic status. Age, head circumference, anthropometric Z-scores and birth weights were significantly different between malnourished and nourished (*P* < 0.001), while sex and WAMI index did not show difference between these groups.

**Table 3:** Characteristics of the malnourished and nourished study population positive for EAEC: age, sex, birth weight, head circumference, length for age *z*-score, weight for age *z*-score, weight for length and Water and sanitation, Maternal education, and Income (WAMI index).

Characteristics	Total	Nourished	Malnourished	*P* values#
	N = 125	N = 59	N = 66	
Age (months; mean ± SD)	12.75 ± 5.38	10.42 ± 4.43	14.83 ± 5.34	<0.0001
Male	59 (47%)	24 (40%)	35 (53%)	0.2096
Birth weight (kg; mean ± SD)	2.86 ± 0.81	3.19 ± 0.66	2.57 ± 0.83	<0.0001
LAZ^1^ (mean ± SD)	−1.67 ± 1.53	−0.48 ± 1.05	−2.74 ± 1.02	<0.0001
WAZ^2^ (mean ± SD)	−1.22 ± 1.81	0.427 ± 1.10	−2.71 ± 0.68	<0.0001
WLZ^3^ (mean ± SD)	−0.46 ± 1.62	0.93 ± 1.05	−1.71 ± 0.84	<0.0001
Current head circumference (cm; mean ± SD)	44.46 ± 2.38	45.10 ± 2.01	43.89 ± 2.56	0.0107
WAMI^4^	0.60 ± 0.09	0.61 ± 0.09	0.59 ± 0.10	0.4948

1 LAZ = length for age *z*-score.

2 WAZ = weight for age *z*-score.

3 WLZ = weight for length for age *z*-score.

4 WAMI index = standardized household socioeconomic score calculated accounting variables as improved water and sanitation, maternal education and monthly household income (range 0–1).

# *P* values obtained from Mann–Whitney and chi-square tests, as appropriate.

### 3.2 Prevalence of EAEC VRGs

Besides the 2 genes used for diagnosis, we also investigated the prevalence of 20 other VRGs associated with EAEC pathogenesis. All 125 EAEC positive samples carried at least one sequence encoding VRG. The most common were samples carrying 11 VRGs (17.60%, 22/125), followed by the samples that presented 12 VRGs (14.40%, 18/125). The most prevalent VRG was *eilA* (78.4%, 98/125) that encodes Salmonella HilA homologue, followed by *agg3/4C* (76.80%, 96/125) that encodes the usher for fimbria III/IV, *capU* (75.20%, 94/125) that encodes the hemosyltransferase, *aar* (68.80%, 86/125), which encodes *aggR*-activated regulator, and *app* (64.80%, 81/125) that codifies dispersin. The least frequent sequence was the plasmid encoded AAF/III fimbrial subunit *agg3A* (5.60%, 7/125). The complete list of prevalence of EAEC VRGs can be seen in Table 4.

**Table 4:** Prevalence of enteroaggregative *Escherichia coli* (EAEC) virulence-related genes (VRGs) among case and control children.

EAEC virulence genes	Total – n = 125 (%)	N^o^ of malnourished – n = 66 (%)	N^o^ of nourished – n = 59 (%)
*astA* – aggregative heat-stable toxin A, EAST1	75 (60.0)	40 (60.6)	35 (59.3)
*pet* – plasmid-encoded toxin	31 (24.8)	18 (27.2)	13 (22.0)
*sigA – Shigella* IgA-like protease homolog⁎	49 (39.2)	28 (42.4)	21 (35.5)
*pic* – protein involved in colonization⁎	70 (56.0)	37 (56.1)	33 (55.9)
*sat* – secreted extracelular protease	63 (50.4)	34 (51.5)	29 (49.1)
*orf3* – cryptic protein	77 (61.6)	40 (60.6)	37 (62.7)
*aap* – anti-aggregation protein, dispersin	81 (64.8)	47 (71.2)	34 (57.6)
*agg3A* – AAF/III fimbrial subunit	7 (5.6)	3 (4.5)	4 (6.7)
*sepA* – *Shigella* extracelular protease	49 (39.2)	24 (36.3)	25 (42.3)
*eilA* – *Salmonella* HilA homolog⁎	98 (78.4)	50 (75.7)	48 (81.3)
*aafA* – AAF/II fimbrial unit	35 (28.0)	19 (28.7)	16 (27.1)
*agg3/4C* – Usher, AAF/III-IV assembly unit	96 (76.8)	49 (74.2)	47 (79.6)
*aafC* – Usher, AAF/II assembly#	14 (11.2)	12 (18.1)	2 (3.3)
*aar* – negative regulator of *aggR*	86 (68.8)	45 (68.1)	41 (69.4)
*aggA* – AAF/I fimbrial unit	58 (46.4)	31 (46.9)	27 (45.7)
*capU* – Hexosyltransferase homolog	94 (75.2)	50 (75.7)	44 (74.5)
*air* – Enteroaggregative immunoglobulin repeat protein	52 (41.6)	23 (34.8)	29 (49.1)
*agg4A* – AAF/IV fimbrial unit	48 (38.4)	27 (40.9)	21 (35.5)
*shiA* – shiA-like inflammation suppressor	72 (57.6)	40 (60.6)	32 (54.2)
*aggR* – aggregative adherence regulator	74 (59.2)	41 (62.1)	33 (55.9)

# *P* = 0.0101 after Fisher's exact test.

⁎ EAEC chromosomal genes.

Considering the frequencies of all VRGs, the gene that encodes the usher for fimbria AAF/II (*aafC*) was the only one significantly associated with malnourished (*P* = 0.0101, OR = 6.33 and 95% Confidence Interval = 1.35–29.63).

Based on this association, further investigation of the fimbriae distribution was as follows. The prevalence of the genes *aggA*, *aafA*, *agg3A* and *agg4A* that respectively codify EAEC fimbria subtypes I, II, III and IV was 46.40% (58/125), 28.00% (35/125), 5.60% (7/125), 38.40% (48/125). However, 33.60% (42/125) of the samples did not harbor genes for any of the fimbriae. Four of those 42 samples harbored the genes *aafC* and *agg3/4C*, the both usher assembly investigated by this work. In addition, 40/42 (95.24%) presented the gene *agg3/4C*. However, only 2 samples did not show any fimbria subunit or usher assembly.

Among the 125 samples positives for EAEC, 37 (29.60%) presented only one subtype of fimbria, but 27 (21.60%) and 19 (15.20%) showed 2 or 3 different fimbria subunits, respectively. For the samples showing 2 fimbriae, the most prevalent combination was subtypes I and IV (62.96%). Almost all the samples that presented 3 different fimbriae showed the combination of subtypes I, II and IV (94.73%). None of the different combination of fimbriae subtypes was associated with malnourished or nourished (*P* > 0.05).

### 3.3 Combination of VRGs

In order to verify if there was a certain combination of EAEC VRGs that could be associated with malnourished or control children we employed the CART analysis. We compared the combinations of EAEC VRGs from positive samples presenting both diagnostic genes (*aaiC* + *aatA*) among the WAZ values (n = 61). This choice was based on the fact that only the samples presenting both genes were statistically associated with malnourished children.

**Fig. 1:**
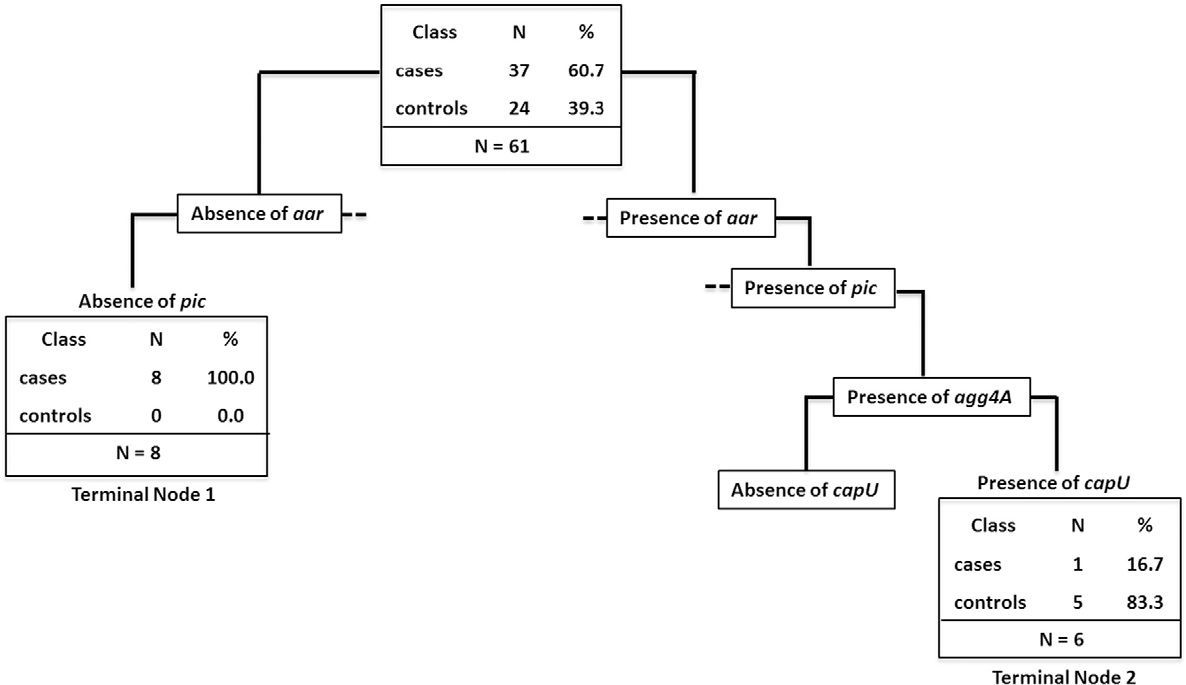
Representative image of the classification tree topology (CART) analysis that shows combinations of EAEC virulence genes most associated to malnourished and nourished children. The tree is hierarchical in nature. Each tree branch ends in a terminal node defined by the presence or absence of the virulence genes in which statistical analysis was performed. Statistical significance (*P* < 0.05) was found only on the terminal nodes 1 and 2. The branches that ended in a non-statistical terminal node were not shown, but represented by dashed lines (− −).

Fig. 1 shows the CART analysis for EAEC positive children based on WAZ. There was one set of cluster ended by terminal node 1 that was associated with malnourished kids and another combination set that was associated with nourished ended by terminal node 2. Samples at node 1 lacked *aar* and *pic* (*P* = 0.018, OR = 14.12, 95% CI = 0.78–257.3). Terminal node 2 showed samples that harbored *aar*, *pic*, *agg4A* and *capU* (*P* = 0.031, OR = 0.11, 95% CI = 0.01–0.97).

### 3.4 Co-infection analysis

In addition, the association of the 61 samples (positive for both *aaiC* and *aatA*) with malnutrition was further analyzed for co-infection. The list of pathogens diagnosed in these samples was described elsewhere (Houpt et al., 2014). Neither pathogen nor other combinations were significantly associated with either cases or controls.

### 3.5 Host biomarkers of intestinal barrier function, intestinal and systemic inflammation

**Fig. 2:**
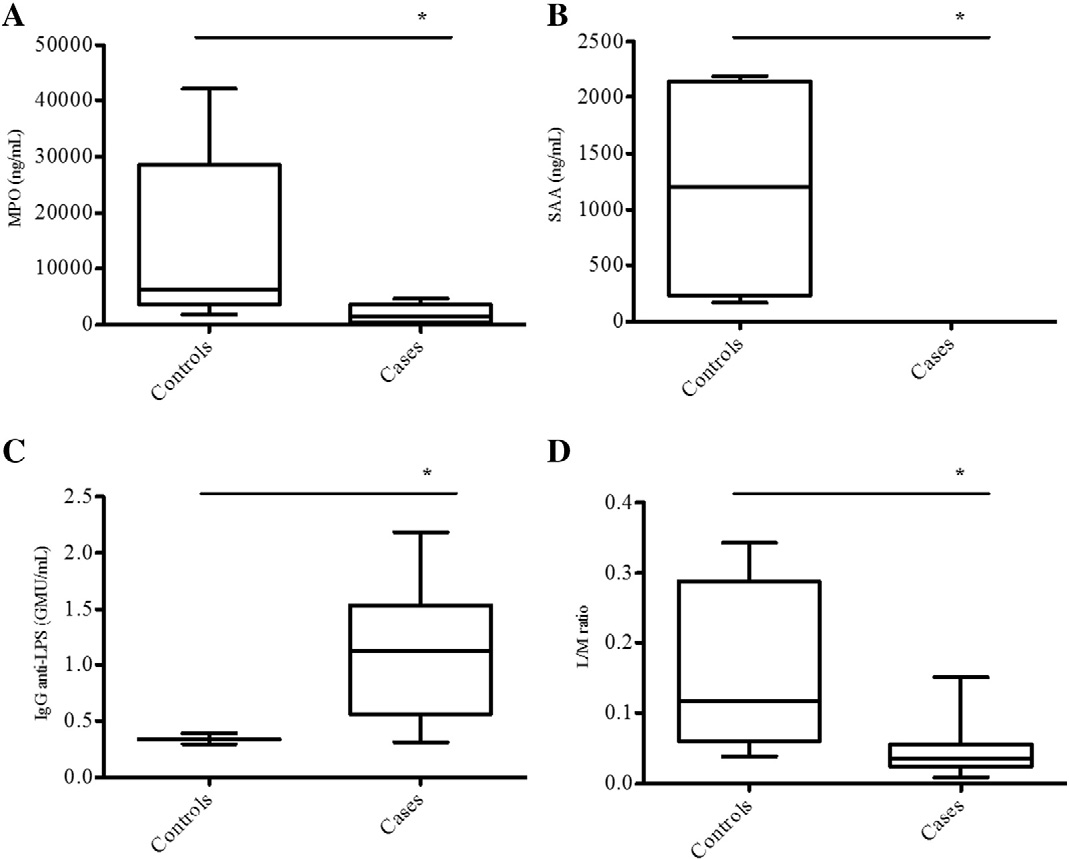
Host biomarkers associated with EAEC-related trait of virulence genes: Myeloperoxidase (MPO) (A), Serum amyloid A (SAA) (B), IgG anti-LPS (C) and lactulose/mannitol ratio (L/M) (D). Children had their specimens collected and frozen at −80 °C pending processing. Fecal MPO and serum SAA and IgG anti-LPS were measured by enzyme linked immunosorbent assays using specific kits, while urinary L/M ratio was measured by high-pressure-liquid chromatography after administration of a solution containing lactulose (250 mg/mL) and mannitol (50 mg/mL) and urine collection. Statistical analysis was performed by Mann–Whitney *U* test (n = 8 for malnourished and n = 5 for nourished). * Significantly different compared to Nourished, *P* = 0.0109 (MPO), *P* = 0.0054 (SAA), *P* = 0.0485 (IgG anti-LPS) and *P* = 0.0480 (L/M ratio).

Children colonized with strains harboring gene combinations associated with nutritional status were assessed regarding the status of selected biomarkers of intestinal barrier function and intestinal and systemic inflammation. The MPO biomarker was increased in both malnourished and nourished children, however higher concentrations were found in the nourished group (*P* = 0.0109). Similarly, higher concentrations of SAA (*P* = 0.0054) and L/M ratio (*P* = 0.0480) were observed in the nourished children. However, malnourished children showed higher concentrations of IgG anti-LPS (*P* = 0.0485) (Fig. 2). All other biomarkers assessed did not vary between groups (*P* > 0.05).

## 4 Discussion

The different outcomes associated with EAEC infection, ranging from acute and persistent diarrhea to subclinical conditions, are a challenge for understanding its pathobiology and proposing therapeutic approaches (Jensen et al., 2014). In this context, the high heterogeneity of EAEC strains is a major contributor and, yet, little is known about the specific virulence markers responsible for these consequences (Chattaway et al., 2013). To our knowledge, this is the first study investigating EAEC virulence factors associated with malnutrition in children 6–24 months old.

The high prevalence of EAEC found in the study population (39.94%), based on the presence of either *aaiC* or *aatA* genes, corroborates with other studies that employed one selected virulence marker for EAEC diagnostic definition among subjects without diarrhea (Bafandeh et al., 2015, Lima et al., 2013, Pereira et al., 2007). Indeed, despite its great prevalence, EAEC has not been associated with diarrhea in recent multi-center cohort studies evaluating children from low and middle-income countries (Kotloff et al., 2013, Platts-Mills et al., 2015).

The concomitant presence of *aaiC* and *aatA* genes was associated with malnourished (WAZ < −2). Chattaway et al. (2013) suggested that the presence of VRGs from within and outside the plasmid AA is necessary for the complete EAEC virulence ability. The diagnosis of EAEC without overt diarrhea has already been associated with HAZ decrements and chronic inflammation in children from Brazil (Steiner et al., 1998). Furthermore, *in vivo* studies have shown that malnutrition increases susceptibility to EAEC colonization (Bolick et al., 2013). The exact mechanisms underlying malnutrition associated with EAEC remain unclear, but impaired intestinal barrier function, intestinal and systemic inflammation might be involved.

Several studies have assessed EAEC virulence factors associated with acute or persistent diarrhea by employing nucleic acid based techniques (Bafandeh et al., 2015, Lima et al., 2013, Nuesch-Inderbinen et al., 2013). The results vary depending on geographic regions and due to the highly heterogeneous EAEC biology. The lack of a specific diagnosis algorithm for EAEC is highlighted in the literature. In order to solve this problem, some authors had proposed an international epidemiologic surveillance for the establishment of a combination of VRGs with biological importance, which can be used as markers for relevant clinical conditions (Boisen et al., 2008, Jensen et al., 2014). Here, a wide panel of 5 multiplex-PCRs was employed for the detection of 20 EAEC VRGs, aiming to identify potential markers of this bacterium and their possible association with malnutrition status in children from Fortaleza, Brazil. In addition, CART analysis was used to evaluate combinations of these different virulence markers associated with undernutrition. This approach has been used in other EAEC studies and is a useful tool for investigating the complex VRGs regulation of EAEC pathogenesis (Boisen et al., 2012, Lima et al., 2013).

The general high prevalence of virulence markers in this study is in agreement with other studies that evaluated subjects without diarrhea, which have shown that strains found among asymptomatic children also presented a high prevalence of VRGs (Bafandeh et al., 2015, Boisen et al., 2012, Ito et al., 2014, Lima et al., 2013, Nuesch-Inderbinen et al., 2013, Regua-Mangia et al., 2009). In addition, this study population did show a low prevalence of diarrhea with no significant correlation with either case or control groups.

AAFs are a major component for EAEC pathogenesis, helping the microorganism to colonize the intestine (Chattaway et al., 2013). In this sense, an overall high prevalence of AAF genes was observed in this study population. The *aafC* gene, encoding the usher for AAF/II assembly, was the only gene associated with malnutrition when the analysis was performed separately, without combination with other genes. AAF/II is not commonly associated with diarrhea (Boisen et al., 2012, Regua-Mangia et al., 2009), except for the studies performed in Nigeria and Brazil that have found that the *aafA* gene, which encodes the AAF/II fimbrial unit, was associated with diarrhea (Lima et al., 2013, Okeke et al., 2000).

The differences observed between the prevalence of fimbrial genes and their corresponding fimbrial usher genes in this study indicate a genetic similarity of these regions within the subtypes of fimbriae and suggest that nearly all EAEC strains harbor an AAF adhesin. Importantly, the usher is a key element for fimbrial biogenesis on facilitating the final assembly of the fimbrial structural organelle (Wurpel et al., 2013). Therefore, these genes have been recently investigated as important virulence markers of EAEC (Boisen et al., 2012, Lima et al., 2013, Nuesch-Inderbinen et al., 2013). In this work, the *aafC* gene had a small prevalence (11.20%), in agreement with other studies that investigated this gene (Boisen et al., 2012, Lima et al., 2013), despite the higher prevalence of the *aafA* gene (28.00%). Interestingly, the previous study of our group done in Fortaleza-Brazil has shown association of *aafA* with diarrhea (Lima et al., 2013). These data indicate a major role of AAF/II for EAEC infection in this population, suggesting association with more severe outcomes.

A higher prevalence of *aggA* (46.4%) observed in this study comparing to the control subjects from a previous case–control study of diarrhea in the same locality (23.5%) suggests a possible importance of this gene in non-diarrheagenic EAEC strains (Lima et al., 2013). Interestingly, a case–control study of diarrhea in Mali has associated *aggA* with subjects without diarrhea (Boisen et al., 2012). Conversely, *agg4A* gene showed a high prevalence of 38.40%, contrasting with previous studies that ranged from 1.6% to 28% (Boisen et al., 2012, Ito et al., 2014, Lima et al., 2013). This gene was associated with control subjects in our previous case–control study of diarrhea (Lima et al., 2013). When evaluating combination of genes associated with undernutrition by CART analysis, *agg4A* was in a combination correlated with nourished. The *agg3/4C* gene, encoding the usher for AAF/III and AAF/IV, had a high prevalence of 76.80%, greater than found in previous studies (Boisen et al., 2012, Lima et al., 2013). Considering the low prevalence of the AAF/III and AAF/IV fimbrial unit genes, *agg3A* and *agg4A*, compared to their related usher *agg3/4C*, this result can be explained by a genetic similarity of this usher with the one from the more recently described AAF/V fimbria (Jonsson et al., 2015). Corroborating to our data, the study of Nuesch-Inderbinen et al. (2013) associated the *agg3C* gene with subjects without diarrhea. Overall, these genes (*aggA*, *agg4A* and *agg3/4C*) may be linked with less severe conditions, regardless of the type of case–control studies.

In this study, strains lacking *aar* and *pic* genes were associated with malnourished children, while the concomitant presence of *aar*, *pic*, *agg4A* and *capU* genes was associated with nourished. The association of the *aar* gene, previously called *orf61*, with non-diarrheagenic strains has been recently shown by 2 case–control studies of diarrhea (Boisen et al., 2012, Lima et al., 2013). In addition, the high prevalence found in the control subjects from these studies was in agreement with the rates found here (68.80%). The *aar* gene is a negative regulator of the major virulence transcriptional factor *aggR* (Santiago et al., 2014). The findings here presented on *aar* reinforce the association of this gene with less severe EAEC outcomes and further investigation on its prevalence in more settings is needed.

The *pic* gene is commonly not associated with diarrhea in clinical studies (Boisen et al., 2012, Lima et al., 2013, Nuesch-Inderbinen et al., 2013, Regua-Mangia et al., 2009) and its function has been suggested to favor intestinal colonization, leading to both mucus hypersecretion and mucinolytic activity (Navarro-Garcia et al., 2010). More recently, Pic was shown to target complement molecules and help immune evasion (Abreu et al., 2015). Importantly, *pic* gene was associated with nourished only in the presence of *aar*. Despite not being controlled by AggR regulator, *pic* expression could be linked with *aar* expression. The possible role of *pic*, in the presence of other selected virulence markers, on protection against malnutrition should be addressed in future studies.

Several studies have associated bacterial virulence with carbohydrates metabolism for *E. coli* and other microorganisms (Somerville and Proctor, 2009). In this context, the *capU* gene, which codifies a hexosyltransferase homolog (Czeczulin et al., 1999), was in the genes combination associated with nourished children. Another study that addressed energy metabolism and bacterial virulence suggested the importance of iron utilization genes for EAEC pathogenesis (Okeke et al., 2004). More studies should be done to question how environmental factors, such as nutritional conditions, influences EAEC virulence mechanisms through metabolism pathways.

Although the virulence genes detected in this study could not be assumed to belong to single bacteria, it is important to note that among the samples included in the CART analysis, 70.49% (43/61) contained only EAEC bacteria (data not shown), which strengthens the conclusions regarding the biological relevance of EAEC and specific virulence factors for malnutrition. Further, the possibility of assessing multiple EAEC bacterial colonies at once could help properly understand the EAEC effects on host that, in such environmental conditions, is certainly colonized with more than one EAEC strain at a time. Indeed, the presence of more than one type of fimbriae in one sample from the study population corroborates to this phenomenon.

This study provided additional knowledge for EAEC pathobiology correlating malnutrition associated EAEC virulence genes with alterations of host biomarkers. Malnourished children infected with strains harboring *aaiC* and *aatA* but lacking *aar* and *pic* genes showed decreased levels of MPO when compared to nourished children infected with strains harboring *aaiC*, *aatA*, *aar*, *pic*, *agg4A* and *capU* genes, indicating lower intestinal inflammatory response. In addition, SAA concentrations were also decreased in these children, corroborating to a less vigorous immune response when compared to nourished group. The higher L/M ratio in the nourished group is due to lactulose permeation, which reflects increased intestinal permeability. In addition, higher levels of IgG anti-LPS in malnourished children indicate more translocation of bacterial products, possibly from EAEC. These data suggest that infection by EAEC strains with specific virulence factors might be correlated with alterations on inflammatory immune response in malnourished children without diarrhea.

This study has provided important knowledge for the understanding of the complex EAEC pathobiology, identifying malnutrition-contributing virulence markers of this pathogen. Our data reinforce the EAEC impact on malnutrition, the importance of *aar* as a negative regulator and the importance of fimbria AAF/II for the pathobiology of EAEC.

## Funding statement

This work was supported in part by the Bill and Melinda Gates Foundation case–control component of the “Etiology, Risk Factors and Interactions of Enteric Infections and Malnutrition and the Consequences for Child Health and Development” Project (MAL-ED), and in part by the collaborative Biomarker Grants No. OPP1066140 entitled, “Novel metabonomic biomarkers of gut function and health: Modeling enteropathy (EE) and field validation”, also supported by the Bill & Melinda Gates Foundation, the Foundation for the NIH, and the National Institutes of Health, Fogarty International Center; The Conselho Nacional de Desenvolvimento Científico e Tecnológico (CNPq Grants: 503,442/2008–9 and 573,928/2008–8).
